# Disease modelling in human organoids

**DOI:** 10.1242/dmm.039347

**Published:** 2019-07-29

**Authors:** Madeline A. Lancaster, Meritxell Huch

**Affiliations:** 1 MRC Laboratory of Molecular Biology, Cambridge Biomedical Campus, Francis Crick Avenue, Cambridge CB2 0QH, UK; 2 The Gurdon Institute, University of Cambridge, Cambridge CB2 1QN, UK; 3 Department of Physiology, Development and Neuroscience, University of Cambridge, Cambridge CB2 3EL, UK; 4 Max Planck Institute of Molecular Cell Biology and Genetics, Pfotenhauerstraße 108, 01307 Dresden, Germany

**Keywords:** Stem cells, Embryonic development, Regenerative medicine, *In vitro*

## Abstract

The past decade has seen an explosion in the field of *in vitro* disease modelling, in particular the development of organoids. These self-organizing tissues derived from stem cells provide a unique system to examine mechanisms ranging from organ development to homeostasis and disease. Because organoids develop according to intrinsic developmental programmes, the resultant tissue morphology recapitulates organ architecture with remarkable fidelity. Furthermore, the fact that these tissues can be derived from human progenitors allows for the study of uniquely human processes and disorders. This article and accompanying poster highlight the currently available methods, particularly those aimed at modelling human biology, and provide an overview of their capabilities and limitations. We also speculate on possible future technological advances that have the potential for great strides in both disease modelling and future regenerative strategies.

## Abstract (precis)

**Summary:** Human organoids are important tools for modelling disease. This At a Glance article summarises the current organoid models of several human diseases, and discusses future prospects for these technologies.

## Introduction

Organoids (see Box 1) are a powerful new system and are being increasingly adopted for a wide range of studies. Indeed, since 2009, over 3000 papers have been published that make use of organoids in some form or another (see poster and Box 2). Organoids can be derived either from pluripotent stem cells (PSCs), adult-tissue-resident cells (stem or differentiated cells) or embryonic progenitors (Huch and Koo, 2015). Because organoids follow the same basic intrinsic patterning events as the organ itself, they are a useful tool for investigating developmental organogenesis or processes of adult repair and homeostasis. Their more accurate organ-like organization makes them a valuable tool for disease modelling, although further improvements, particularly in scalability, are still needed. Nonetheless, because organoids have such potential, extensive effort has been made at generating organoids for a range of organ types. We describe the methods and applications for these in more detail in this article and in the accompanying poster, and discuss future directions for improving this technology and furthering its applications.

**Figure:**
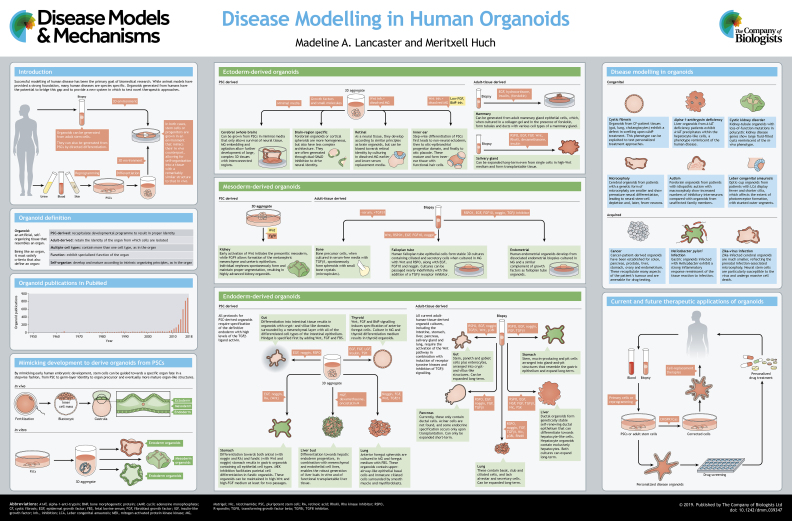

**Box 1. Organoids: definition and applications**

The term ‘organoid’ is actually not new. A simple PubMed search reveals its first usage in 1946 in reference to a tumour case study (Smith and Cochrane, 1946). However, at that time, the term was used to describe certain histological features of tumours, such as glandular organization. Over time, its meaning evolved to generally refer to tissues or structures that resemble an organ, and so this term became increasingly used in *in vitro* biology. However, it wasn't until the development of intestinal organoids in 2009 (Sato et al., 2009) that it began to be used more specifically for these self-organizing *in vitro* structures. Nonetheless, the term ‘organoid’ continues to be used for a wide variety of tissues or structures that may or may not fully recapitulate key features of an organ.

Therefore, in an attempt to clarify some of the confusion surrounding this term, we use a previously proposed working definition (Lancaster and Knoblich, 2014; Huch and Koo, 2015; Clevers, 2016) that fulfils the most basic definition; namely, ‘resembling an organ’. More specifically, a genuine organoid should satisfy several criteria: (1) a 3D structure containing cells that establish or retain the identity of the organ being modelled; (2) the presence of multiple cell types, as in the organ itself; (3) the tissue exhibits some aspect of the specialized function of the organ; and (4) self-organization according to the same intrinsic organizing principles as in the organ itself (Sasai, 2013a,b).

**Box 2. Birth of the organoid field**

Since the dawn of biology as a scientific field, scientists have sought to understand mechanisms of human disease. While studying patients can give insight into symptoms and help describe the course of the disease, the underlying causes are often enigmatic. Therefore, animal models have been a staple of human disease research, as they can be engineered to develop the disease, for example, by introducing relevant mutations. However, because of evolutionary divergence, there are many features of human biology and disease mechanisms that cannot be accurately modelled in animals. For instance, rodent models of Alzheimer's disease do not ubiquitously display the characteristic plaques and tangles (Asaad and Lee, 2018). When key disease features are absent from animal models, it is difficult to study the underlying mechanisms.

For these reasons, efforts over the past several decades have focused on modelling human disease biology in a dish. While HeLa cells and other immortalized human cell lines have proven to be a powerful tool, their use has several considerable drawbacks, including their genomic instability and limited tissue identities (Adey et al., 2013). Furthermore, the expansion of adult primary tissues far beyond the predicted Hayflick limit is a real challenge (Hayflick, 1965). Thus, more recent approaches have focused on *in vitro* models derived from stem cells, which allow for a broader array of tissue identities, long-term expansion, better genomic integrity and improved modelling of healthy biology. A stem cell is any cell that is able to self-renew and generate differentiated progeny, i.e. capable of generating several defined identities. Thus, differentiated organ cell types can be generated *in vitro* from pluripotent stem cells (PSCs) by following a series of differentiation steps that mimic early embryonic development. Alternatively, differentiated cells derived from resident adult stem/progenitor cells, such as the stem cells of the intestinal crypt, can be used but historically these have proven difficult to expand *in vitro* (Bjerknes and Cheng, 2006).

In order to overcome these limitations and also better recapitulate tissue architecture, a new field of 3D *in vitro* biology called tissue engineering has come into the spotlight. By combining biology and engineering, more elaborate conformations of cells have been established, allowing for multiple cell types to be combined in a configuration that more closely mimics organ architecture. Perhaps the most exciting developments have been the recent organ-on-a-chip methods, which allow for the construction of connected chambers that mimic different organ compartments, for example liver ducts and blood vasculature (Huh et al., 2011).

While tissue engineering has provided a number of highly useful models for looking at the interaction between different cell types, there are certain artificial aspects that affect their ability to accurately model organ structure and therefore function. For example, the use of artificial membranes and matrices means that the cells are positioned via exogenous processes, rather than through bottom-up self-organizing principles as in organ development *in vivo*. Thus, to more accurately model organ architecture, very recent efforts have focused on supporting the self-organizing development of these tissues *in vitro*. And so, the organoid field was born.

## Pluripotent stem-cell-derived and adult-tissue-derived organoids

One of the major leaps that has led to the development of organoid methods was the realization that, to better recapitulate organ morphology *in vitro*, one must first understand the development of that organ and try to mimic it. Thus, years of research on the patterning events and signalling cascades at play during organogenesis have provided the necessary foundation to make organoid research possible. The relatively recent advent of human pluripotent stem cell (PSC) cultures has provided the starting point for this process.

Remarkably, human PSCs can be induced to spontaneously undergo differentiative and morphogenetic behaviours that mimic the formation of embryonic germ layers, especially when they are forced to form three-dimensional (3D) aggregates called embryoid bodies (EBs; Box 3). EBs form germ layers that express well-described molecular markers and even segregate to form patches of individual germ-layer tissue within the aggregate. By applying specific growth factors, these aggregates can be directed towards a specific germ layer.

**Box 3. Germ-layer formation: the starting point of organogenesis**

All organs develop from primitive tissues that form at the very early onset of embryogenesis. After fertilization, cells within the early blastula establish two main compartments: the extraembryonic tissue, which will give rise to the supportive foetal environment including placenta and amniotic sac, and the inner cell mass (ICM), which will give rise to the embryo proper. Human pluripotent stem cells (PSCs) can be thought of as roughly equivalent to ICM stem cells, and indeed human ESCs are taken from the ICM of the human blastocyst. Upon gastrulation, the embryonic disc, which derives from the ICM, undergoes morphogenetic movements that establish the three germ layers: endoderm, mesoderm and ectoderm.

Progenitors within these germ layers have restricted potentials to generate primordial organ structures, and their specific identity will depend on their spatial context relative to one another, and on where they lie on the anterior-posterior and dorsal-ventral axes (Zernicka-Goetz and Hadjantonakis, 2014). This is due to the early establishment of gradients of morphogens that will influence differentiation towards particular subregions of these germ layers. For example, the endoderm will give rise to the entire gastrointestinal tract and, depending on their location, endodermal progenitors can give rise to more anterior identities, such as the stomach, or to more posterior identities, such as the colon. This is due to an anterior-posterior gradient of signalling factors like Wnt, BMP and FGFs (Zorn and Wells, 2009).

Similarly, BMP4 promotes formation of the mesendoderm, a precursor to certain mesoderm and endoderm types, whereas early activation of Wnt can promote the presomitic mesoderm, the precursor to the kidney (Little et al., 2016). In contrast, in the absence of growth factors or serum, the embryoid body tends to be biased towards the ectoderm as the default. By understanding these specific morphogen gradients and their effects on the primordial tissue, these growth factors can be provided at specific concentrations and with specific timing to direct the differentiation of progenitors towards certain organ identities.

Biologists have taken advantage of the knowledge on developmental events to construct or reconstruct a tissue *in vitro* (organoids) that recapitulates many of the key structural and functional features of the organ (Box 1). The range of organoids established so far is rapidly increasing, and although in many cases these have been first developed from rodent PSCs, researchers are translating experimental methods to human cells.

The use of PSCs as a starting point to generate 3D organoid cultures allows the *in vitro* recapitulation of the developmental processes and tissue organogenesis that occur *in vivo*. However, once organ growth is terminated, adult tissues still require tissue maintenance and repair to ensure proper functionality of the adult organ. Recently, a better understanding of the signalling pathways important for adult tissue maintenance and repair has enabled researchers to grow primary tissues as self-expanding organoids that retain all the characteristics of the tissue of origin, as well as their genetic stability over time (see Huch and Koo, 2015 for an extended review; Table 1).

**Table 1.**
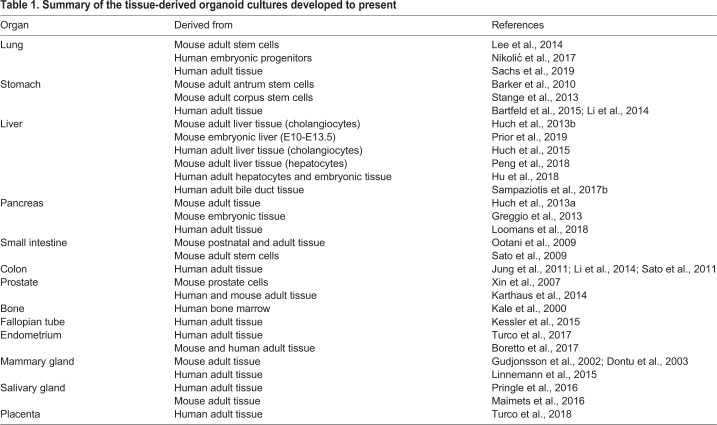
**Summary of the tissue-derived organoid cultures developed to present**

Here, we summarize the methods for derivation of organoids from human cells, be it PSCs or tissue-resident cells.

## Endoderm-derived organoids

The development of 3D organoid cultures has proven very successful for several endoderm-derived organs, such as the intestine, stomach, liver, pancreas and lung. In this section, we discuss the findings and conditions used to develop these organoids.

### Intestinal organoids

In the intestine, Wnt, Notch, fibroblast growth factor (FGF)/epidermal growth factor (EGF) and bone morphogenetic protein (BMP)/Nodal signalling are required during tissue development and in adult homeostasis and repair. By combining the knowledge on stem-cell populations and on the intestinal stem-cell niche requirements, intestinal organoids from either postnatal or adult intestinal epithelium (Ootani et al., 2009) or from a single adult intestinal stem cell (Sato et al., 2009) have been established, with an expansion potential far beyond the Hayflick limit (>1 year in culture). Similarly, mouse- and human-derived colonic stem cells have also been expanded in organoid cultures with minor adjustments in the medium composition (Sato et al., 2011). The culture conditions that support intestinal organoids include embedding the intestinal stem cells (or cells with the ability to acquire stem-cell potential, i.e. immediate daughters of the stem cells) in a 3D extracellular matrix (e.g. Matrigel) and culturing them in a medium supplemented with EGF, noggin and the Wnt agonist R-spondin (RSPO). These conditions allow the long-term expansion of adult intestinal epithelium in culture while retaining its ability to differentiate into individual derivatives (Sato et al., 2011; Ootani et al., 2009).

In parallel studies, the Wells lab successfully differentiated PSCs into gut epithelium *in vitro* by treating definitive endoderm-specified cells with Wnt3a and FGF4 to induce posterior endoderm patterning and hindgut specification (Spence et al., 2011). These conditions result in the formation of spheroids after 2 days. Then, upon transfer into Matrigel and incubation in culture conditions that support the growth of adult-tissue-derived organoids, these PSC-derived spheroids formed bona-fide small-intestinal organoids. Recently, these PSC-derived intestinal organoids have been combined with neural crest cells to recapitulate the normal and functional intestinal enteric nervous system (Workman et al., 2017; Schlieve et al., 2017).

### Gastric organoids

Gastric organoids have been obtained by either expansion of adult gastric stem cells from both the corpus and the antropyloric epithelia, as well as from directed differentiation of PSCs. The identification of Lgr5 as a marker for pyloric stem cells (Barker et al., 2010) led to the development of the first long-term culture system of mouse gastric stem cells. Corpus and pylorus stem cells were grown in Matrigel and in medium supplemented with WNT3A and FGF10 (Barker et al., 2010; Stange et al., 2013). Blockade of transforming growth factor-beta (TGFβ) signalling enabled the expansion of human gastric organoids from both pylorus and corpus epithelium that would contain all gland and pit cell types (Bartfeld et al., 2015). Differentiation into acid-producing parietal cells proved more difficult, though. Only upon co-culture with the mesenchymal niche, corpus organoids from neonatal (Li et al., 2014) and adult (Bertaux-Skeirik et al., 2016) mouse stomach tissue could differentiate into this mature cell type. The human counterpart has not been achieved, yet.

To obtain PSC-derived gastric organoids, McCracken et al., upon establishing definitive endoderm with activin (Kubo et al., 2004; D'Amour et al., 2005), exposed the cells to Wnt, FGF4 and noggin to enable gastric specification. Noggin is essential to prevent intestinalization and to promote a foregut fate (McCracken et al., 2014), while retinoic acid facilitates antrum epithelium specification. Matrigel is essential for the formation of 3D foregut structures. When maintained in an EGF-rich medium, these structures generate gastric organoids that contain all antral epithelium cell types (McCracken et al., 2014). Noguchi and colleagues used mouse PSCs to generate a functional corpus epithelium *in vitro* that contained acid-producing cells (Noguchi et al., 2015). In a different approach, Wells and colleagues observed that, after foregut patterning, sustained Wnt signalling using the GSK3β inhibitor CHIR enabled fundus (corpus) specification instead of antrum. These fundic cells could be subsequently differentiated into all cell types of the gastric epithelium, including functional parietal cells (McCracken et al., 2017). Interestingly, the authors demonstrate that their PSC-differentiated fundic organoids contain bona-fide stomach progenitors that can be isolated, cultured and further propagated in the tissue-derived organoid medium described by Barker, Huch and colleagues (Barker et al., 2010) to expand adult-tissue-derived organoids (McCracken et al., 2017), thus linking, for the first time, the development of both types of organoids.

### Liver organoids

Adult hepatocytes and cholangiocytes (also known as ductal cells) are the two endodermal-derived cell types in the adult liver, yet the organ is composed of mesoderm-derived hepatic mesenchymal cells. Michalopoulos et al. first described liver-derived 3D structures back in 2001. In these studies, these ‘organoids’ were very different from what we now consider liver organoid cultures, as they would only survive for a short period of time in culture, yet they retained some of the function and structure of the hepatocyte epithelium (Michalopoulos et al., 2001). It was not until 2013 that the first liver organoids as we know them now were described. Huch et al. established the first adult murine-tissue-derived liver organoid culture that sustains the long-term expansion of liver cells *in vitro* (Huch et al., 2013b). By combining Matrigel with hepatocyte growth factor (HGF), EGF and liver-damage-induced factors FGF (Takase et al., 2013) and RSPO1 (Huch et al., 2013b), the isolated liver cells self-organized into 3D structures that retained the ability to differentiate into functional hepatocyte-like cells, even when grown from a single cell (Huch et al., 2013b). Addition of an activator of cyclic adenosyl monophosphate (cAMP) signalling and inhibition of TGFβ signalling adapted this culture system to the expansion of adult human liver cells as self-renewing organoids that recapitulate some function of *ex vivo* liver tissue (Huch et al., 2015). Of note, both human and mouse cultures could be established from single cells, hence enabling, for the first time, the study of mutational processes in healthy tissue (Huch et al., 2015; Blokzijl et al., 2016).

Takebe et al. took a completely different approach and, by mixing human induced PSC (iPSC)-derived hepatocytes with mesenchymal stem cells (MSCs) and umbilical cord cells (HUVECs), obtained the first embryonic liver bud organoids formed by proliferating hepatoblasts and supporting cells. When transplanted into different mouse sites, these developed into mature functional hepatic tissue (Takebe et al., 2013). In a follow-up study, Takebe and colleagues also differentiated iPSCs towards the three hepatic progenitors – hepatic endoderm, endothelium and septum transversum mesenchyme – hence overcoming the issue of using postnatal tissue-derived stromal progenitors (Takebe et al., 2017). In 2015, Sampaziotis et al. also generated cholangiocyte organoids from iPSCs (Sampaziotis et al., 2015). Whether combining both protocols could generate structures containing both hepatocytes and cholangiocytes or whether, instead, a better specification of true bi-potent hepatoblasts, like the novel Lgr5+ population described by Prior et al. (2019) is required first to achieve this goal, remains to be determined.

Since these seminal papers, altered protocols have enabled the establishment of liver models from different species from rat to dog (Nantasanti et al., 2015; Kuijk et al., 2016; Kruitwagen et al., 2017), as well as human disease models ranging from alpha-1-antitrypsin  (A1AT) deficiency and Alagille syndrome (Huch et al., 2015) to polycystic liver disease (Wills et al., 2016) or cancer (Broutier et al., 2017; Nuciforo et al., 2018). By modifying the Huch conditions to expand human organoids (Huch et al., 2015), the Vallier lab has recently established extrahepatic biliary organoids and showed that these can form bile-duct-like tubes that can reconstruct the gallbladder wall upon transplantation into mice (Sampaziotis et al., 2017a).

Finally, the Nusse and Clevers labs recently reported the establishment of hepatocyte organoids derived from adult mouse hepatocytes (Peng et al., 2018; Hu et al., 2018), while the Huch lab recently established clonal mouse hepatoblast cultures (Prior et al., 2019). While human adult hepatocyte cultures are yet to be established, human embryonic cultures that excellently recapitulate the bile canaliculi structure *in vitro* have been recently described (Hu et al., 2018). Whether these cultures can differentiate into more mature hepatocyte or cholangiocyte derivatives and/or exhibit the extensive cellular plasticity of the *in vivo* tissue is still to be investigated.

### Pancreatic organoids

Similarly to the liver, both embryonic and/or adult pancreas cells are difficult to expand or maintain in culture. By isolating mouse embryonic pancreas progenitors and culturing them in Matrigel, Grapin-Botton and colleagues elegantly showed that the cells could recapitulate pancreatic embryonic development *in vitro* and generate exocrine (acinar) and endocrine (insulin-producing) cell derivatives (Greggio et al., 2013). By using a similar culture system as the adult liver organoids described above, Huch et al. established the first adult mouse pancreas organoid model by seeding pancreas ductal cells in Matrigel in the presence of FGF10, noggin, RSPO1 and EGF (Huch et al., 2013a). A similar system was later applied to human pancreatic cancer cells (Boj et al., 2015), although long-term expansion of healthy human pancreas tissue is still to be achieved. Recently, Loomans et al. expanded human pancreas tissue for a few passages and obtained structures formed exclusively of ductal epithelium. After transplantation into mice, these expressed some endocrine markers, although did not differentiate into islets (Loomans et al., 2018). Unlike the Grapin-Botton lab's embryonic cultures described above, Loomans' adult-tissue-derived ones cannot differentiate into endocrine or acinar cells *in vitro*. Whether that is because adult pancreas ductal cells have lost their endocrine differentiation potency or whether the external cues have not been identified yet is unknown.

### Lung organoids

The term ‘lung organoids’ describes both upper- and lower-airway organoids. Protocols for both PSC- and adult-tissue-derived organoids have been established. Rossant and colleagues differentiated human iPSCs to lung epithelium using air-liquid interphase culture (Wong et al., 2012). This initial protocol was later modified by the Spence lab to instruct the cells towards a foregut fate by adding TGFβ/BMP inhibitors, FGF4, and Wnt activators. Subsequent activation of the Hedgehog pathway with the Smoothened agonist SAG resulted in lung specification. Transferring these specified 3D spheroids into Matrigel and culturing them in FGF10-rich medium enabled the differentiation to lung organoids containing airway-like structures formed by basal, ciliated and club cells, surrounded by a mesenchymal compartment (Dye et al., 2015). Interestingly, when transplanted in a bioartificial microporous poly(lactide-co-glycolide) scaffold, full maturation and differentiation of secretory lineages was observed, while long-term engraftment enabled these to differentiate into distal lung cells (Dye et al., 2016). Parallel studies by the Snoeck lab also showed the formation of 3D structures that developed into branching airway and early alveolar epithelium after xenotransplantation (Chen et al., 2017).

Establishing organoids from adult lung tissue has been more difficult. Hogan and colleagues pioneered upper-airway organoids, reporting that single basal cells would self-organize into bronchiolar lung organoid cultures. These had limited expansion potential, yet were able to differentiate to both basal and luminal cells (Rock et al., 2009). Building on these cultures, the Rajagopal lab was able to expand airway basal stem cells by inhibiting SMAD signalling (Mou et al., 2016). The generation of distal (lower)-airway lung organoids has been more difficult. Alveoli consist of surfactant-secreting type II (AT2) and gas-exchanging type I (AT1) cells. By identifying and expanding bronchioalveolar stem cells (BASCs), Kim and colleagues showed that single BASCs had bronchiolar and alveolar differentiation potential in lung organoids co-cultured with lung endothelial cells (Lee et al., 2014). Similarly, Barkauskas et al., by co-culturing human AT2 cells with lung fibroblasts, established self-renewing human alveolar organoids (Barkauskas et al., 2013). Co-culturing macrophages with AT2 cells promoted the formation of organoids from AT2 cells (Lechner et al., 2017) in a dose-dependent manner (i.e. the more macrophages the more AT2 growth).

Only recently, two labs have been able to expand lung progenitors in the absence of a mesenchymal or endothelial niche: the Rawlins lab described the first human long-term self-renewing lung organoids derived from embryonic lung (Nikolić et al., 2017), while the Clevers lab generated 3D human airway organoids containing ciliated, goblet, club and basal cells (Sachs et al., 2019). The generation of self-renewing human alveolar organoids in a defined medium still awaits development, though.

### Thyroid

Although it was well known by the mid-20th century that dissociated rat thyroid cells aggregate and reconstitute functionally active thyroid tissue *in vitro* (Mallette and Anthony, 1966), it was not until very recently that researchers successfully generated thyroid organoids from PSCs. This delay was due to the lack of knowledge on how thyroid tissue develops. By studying the transcription factors important for thyroid development, Antonica and colleagues found that forced expression of thyroid-specific transcription factors in PSCs resulted in thyroid cells that formed follicles *in vitro* and upon transplantation (Antonica et al., 2012). More recently, Kotton and colleagues identified the right combination of BMP and FGF signalling to enable the differentiation of iPSCs to a thyroid fate. By isolating these thyroid progenitors and cultivating them in Matrigel, the authors generated follicular organoids consisting of a monolayer of hormone-producing thyroid cells. Of note, following xenotransplantation, these organoids restored TH levels in a hypothyroid mouse model (Kurmann et al., 2015). Whether self-renewing human thyroid organoids can be obtained from adult thyroid epithelium remains to be discovered. In light of the early studies from Mallette and Anthony and the recent report from Nagano and colleagues suggesting that mouse thyroid organoids can be developed from adult tissue (Saito et al., 2018), we speculate that the human counterpart will soon also be developed.

## Mesoderm-derived organoids

The embryonic mesoderm gives rise to a variety of internal organs, including kidney, heart, cartilage, bone, reproductive organs and muscle. We detail below the methods to generate the respective human organoids.

### Renal organoids

Kidney organoids are an ideal illustration of the successful generation of an *in vitro* model based upon careful characterization of *in vivo* development. In particular, several recent studies have begun to shed light on where the key kidney precursors come from. Work from Taguchi et al. (2014) was instrumental in characterizing the early stages of metanephric kidney development, particularly the formation of metanephric mesenchyme (MM), then applying the identified signalling factors to direct differentiation of mouse and human PSCs specifically towards MM cells that could form 3D structures when co-cultured with mouse tissues. This study helped define the developmental steps leading to kidney formation.

While Taguchi et al. demonstrated the successful induction of MM, Takasato et al. (2014) and Xia et al. (2013) described conditions that would also give rise to the ureteric bud (UB). In both studies, the authors initially directed PSCs towards the intermediate mesoderm by first mimicking signalling events involved in primitive streak formation. In the Xia et al. study, the cells then gave rise to UB precursors that could mature and integrate into co-cultures with embryonic mouse kidney. Takasato et al. instead simultaneously generated human UB and MM identities. Not only could these cells integrate into embryonic mouse kidney, but they could also form rudimentary tubule structures upon aggregation even without the supportive mouse tissue.

The variety of approaches for generating early self-organising renal structures generally points to important roles for several growth factors, including BMP4, retinoic acid, FGFs, Wnt and activin A. However, a breakthrough came with the establishment of the entirely self-organizing 3D human renal organoids by Takasato et al. (2015), which involved minimal use of growth factors (just CHIR99021 to activate Wnt, and FGF9), and led to structures with all the major components of the developing kidney, including the collecting duct, proximal and distal tubules, glomeruli, and even endothelial networks.

### Bone spheroids

Although proper bone organoids have not yet been established, Kale et al. described a promising approach to form spheroids of human adult bone precursor cells (Kale et al., 2000). These are a heterogeneous population of bone progenitors, including osteoblasts, that can be induced to form small pieces of crystalline bone called microspicules. This requires 3D aggregation of the cells as well as the removal of serum and addition of TGFβ1. Given that this approach lacks the varied cell types and tissue architecture, it will be interesting to see whether current improvements in 3D culture methods could further develop these spheroids to bona-fide bone organoids.

### Fallopian tube organoids

Adult-derived organoid methodology has recently been applied to the fallopian tube of the female reproductive tract. The method described (Kessler et al., 2015) has many similarities to the Barker and Huch et al. stomach organoid method and involves culture of human adult fallopian tube epithelial cells in Matrigel in the presence of mouse gastric organoid medium (Barker et al., 2010) with TGFβ inhibition. The resulting 3D cystic spheres develop various invaginations as they mature, and contain both ciliated and secretory cells. These tissues also respond to the sex hormones estradiol and progesterone in a manner reminiscent of the response of this tissue *in vivo* during the menstrual cycle.

### Endometrial organoids

Two independent groups have derived organoids from the adult endometrium using similar conditions as those described for adult liver organoids (Turco et al., 2017; Boretto et al., 2017). Hence, these required the presence of RSPO, EGF, FGF10 and noggin, but no WNT supplementation. Both studies demonstrated that the endometrial organoids respond to estradiol and progesterone in specific and differential manners. Specifically, estradiol stimulated increased proliferation while progesterone stimulated a more mature morphology, mimicking the later secretory phase of the menstrual cycle. These morphological and transcriptomic changes recapitulated the changes in the endometrium during the menstrual cycle. Furthermore, Turco et al. exposed the endometrial organoids to pregnancy signals (prolactin, human chorionic gonadotropin and human placental lactogen), which led to a decidua-like morphology similar to that seen in early pregnancy.

## Ectoderm-derived organoids

The embryonic ectoderm gives rise to two main tissue types: surface ectoderm, which will develop into skin and its associated glands and hair; and neural ectoderm, which will give rise to the brain, spinal cord and neural crest. The neural crest is a highly multipotent entity that can further differentiate into the peripheral nervous system, as well as bone, cartilage, connective tissue, and vasculature of the head, and even contributes to the heart.

### Mammary organoids

For decades, mammary cells have been cultured in 3D extracellular matrix gels as a model for mammary gland development, homeostasis and tumorigenesis (Hall et al., 1982). Indeed, isolated mouse mammary epithelial cells were some of the first cells to be cultured in Matrigel (Li et al., 1987), and could give rise to branched structures reminiscent of the mammary gland. However, differences between mouse and human mammary stroma have made it difficult to directly translate these methodologies to a human *in vitro* model.

The first 3D branched mammary-gland-like structures from human mammary cells were described by Gudjonsson et al. (2002), who isolated a progenitor population that could generate several cell types and the typical branched morphology of the mammary gland when embedded in Matrigel. Similarly, Dontu et al. described the formation of mature ductal-acinar-like structures from multipotent human mammary epithelial cells upon embedding in Matrigel and exposure to prolactin (Dontu et al., 2003). Importantly, these studies successfully generated mammary-gland-like organoids from cells first expanded *in vitro* either as a cell line or in mammospheres.

More recently, researchers devised methods to generate branching structures directly from isolated human mammary epithelial cells (Linnemann et al., 2015). The formation of these structures similarly relies upon embedding in 3D matrix gels and the presence of growth factors, including EGF, hydrocortisone and insulin, to promote mammary epithelial-stem-cell proliferation. Under these conditions, and with the addition of forskolin (Fsk), human mammary epithelial cells spontaneously generated structures reminiscent of the terminal ductal lobular units of the mammary gland. Importantly, these structures contained multiple cell types at correct positions within the branched structure and showed contractile activity, a feature of the basal/myoepithelial cells that eject milk during lactation.

### Salivary gland

There is some controversy about the developmental origin of the salivary glands – whether ectodermal or endodermal (see Patel and Hoffman, 2014 and de Paula et al., 2017 for details), yet mouse lineage-tracing studies suggest an ectodermal origin (Rothova et al., 2012), although there is no direct evidence for this yet. By applying the Clevers organoid method to primary adult salivary gland cells, Coppes and colleagues efficiently generated long-term-expanding organoids from the mouse (Maimets et al., 2016) and human (Pringle et al., 2016) salivary gland. The culture conditions required high levels of Wnt activation to obtain organoids containing all differentiated salivary gland cell types. Transplantation of these organoids into murine submandibular glands restored saliva secretion and increased the number of functional salivary gland acini *in vivo* (Pringle et al., 2016). These studies hold the promise of salivary gland transplantation as a potential treatment for xerostomia (severe hyposalivation).

### Retinal organoids

The retina is the neural part of the eye and contains photoreceptors, supportive cells and interneurons. Self-organizing retinal tissues were the first entirely 3D neural organoids to be successfully established from mouse (Eiraku et al., 2011) and later human (Nakano et al., 2012) ESCs. As with many organoids, Matrigel turned out to be a key component. Both studies described a very minimal medium, along with Wnt inhibition, to develop retinal identities from ESCs. But it was the addition of dissolved Matrigel that allowed for the formation of a more rigid epithelium that could adopt the morphology of the retinal primordium, the optic cup. These optic-cup-like organoids begin as aggregates containing large spherical vesicles of neuroepithelium with an early retinal identity and appear similar to optic vesicles. As development proceeds, these vesicles spontaneously invaginate to from a cup-like morphology reminiscent of the developing optic cup.

Optic-cup organoids form the typical stratified architecture of the developing retina, including rods and cones. However, generating functional photoreceptors, including light-sensitive outer segments, has been a challenge. Since the initial description of self-organizing retinal tissues from human ESCs, others have improved upon these methods and recently described extended culture times and further maturation of the photoreceptors to include rudimentary outer-segment discs and even occasional light responsiveness (Wahlin et al., 2017).

### Brain organoids

*In vitro* modelling of human brain development has been a fast-evolving field that builds upon decades of *in vivo* and *ex vivo* work. The discovery in 2001 that human ESCs could form 2D rosette-like structures after an initial phase of 3D culture as an EB (Zhang et al., 2001) revealed the self-organization potential of neural progenitors to form neural-tube-like structures. Eiraku et al. then expanded upon this by extending the initial 3D culture phase before plating on coated dishes, which allowed the formation of even more complex stratified structures reminiscent of the developing cerebral cortex (Eiraku et al., 2008; Mariani et al., 2012). Specifically, neural progenitors could self-organize to form a continuous neuroepithelium, similar to retinal organoids, but as these brain-regionalized structures developed, progenitors generated neurons that properly migrated away from germinal zones to populate a more basal region reminiscent of the pre-plate of the cerebral cortex. This germinal zoning and segregation of post-mitotic neurons is highly reminiscent of the developing human brain.

These initial cultures used dual SMAD and Wnt inhibition (Watanabe et al., 2005; Eiraku et al., 2008) to promote the formation of relatively pure neural identities as well as to direct differentiation towards a telencephalic fate. Subsequent work showed that, in the absence of these inhibitors, simply providing a very minimal medium to prevent the expansion of non-neuroectodermal identities could generate a more broad brain regional identity (Lancaster et al., 2013). Furthermore, embedding in Matrigel led to a dramatic reorganization and expansion of the neuroepithelium that allowed the formation of neural-tube-like buds that further expanded without the need for replating the aggregates. Like the telencephalic structures described above, these cerebral organoids developed cortical regions with characteristic germinal and differentiated zones. However, these 3D tissues also formed a variety of brain regions, from hindbrain to retinal identities, within the same organoid. Similarly, maintenance as a floating aggregate was also applied to the telencephalic structures to generate 3D forebrain organoids with improved tissue architecture (Kadoshima et al., 2013).

Since the establishment of telencephalic and cerebral organoids, subsequent studies further modified these approaches and generated organoids with more specific brain regional identities, including cortical spheroids (Pas¸ca et al., 2015), hippocampal organoids (Sakaguchi et al., 2015), midbrain organoids (Jo et al., 2016), pituitary and hypothalamic organoids (Suga et al., 2011; Ozone et al., 2016), and cerebellar organoids (Muguruma et al., 2015). More recently, researchers fused regionalized brain organoids, revealing that interneurons generated within the ventral telencephalic region could indeed migrate to dorsal cortical tissue as they do *in vivo* (Birey et al., 2017; Bagley et al., 2017; Xiang et al., 2017). Finally, extended culture of cerebral organoids resulted in neuron maturation within the organoid, allowing for the formation of rudimentary networks and even rare responses to light in these whole-brain organoids containing retinal cells (Quadrato et al., 2017).

### Inner-ear organoids

The inner ear develops from non-neural ectoderm and contains thousands of hair cells that respond to minute air vibrations and thus allow us to hear, as well as detecting head movements and gravity. Koehler et al. developed human inner-ear organoids from ESCs by using Bmp4 to promote the non-neural ectoderm lineage as opposed to the neural ectoderm (Koehler et al., 2017). However, TGFβ inhibition prevented unwanted mesoderm and endoderm formation. This approach led to the successful formation of an epithelium on the surface of 3D aggregates of ESCs that expressed otic placode markers, the precursor to the inner ear. Wnt activation subsequently led to the development of otic vesicles with supportive mesenchyme and later formation of epithelia-containing sensory hair-like cells.

## Disease modelling in organoids

A primary goal of human organoids is their use in modelling human diseases in order to establish paradigms for drug screening, genotype-phenotype testing and even biobanking for specific diseases and future personalized treatments, including cell therapies. While the establishment of a human organoid model for most organs is still quite new, and therefore disease modelling in this manner is still in its infancy, there have already been several examples of using organoid cultures to study congenital or acquired human diseases.

### Congenital conditions

The first human condition to be modelled in organoids was cystic fibrosis (CF), in the intestinal organoid system. CF is caused by mutations in the cystic fibrosis transmembrane conductance regulator (CFTR) chloride channel, which is normally expressed in epithelial cells of many organs. Dekkers and colleagues (2013) generated CF-patient-derived intestinal organoids that could recapitulate the disease *in vitro*. They developed a swelling assay in which wild-type organoids respond to the activation of cAMP by importing fluid to the lumen and subsequently swelling, whereas this response is abolished in CF organoids. This approach demonstrated the excellent predictive value of intestinal organoids for identification of responders to CFTR modulators, and has become the first personalized treatment test for CF patients in The Netherlands (Berkers et al., 2019). In a parallel approach, Vallier and colleagues also showed that iPSCs from CF patients differentiated into liver cholangiocytes can also model CF *in vitro*, as these cells also lack the ability to swell compared to wild-type controls (Sampaziotis et al., 2015). In addition, Huch et al. showed that liver organoids from patients with A1AT deficiency recapitulated the epithelial features of the disease, whereby precipitates of the misfolded A1AT protein accumulated in the differentiated hepatocyte-like cells *in vitro* (Huch et al., 2015). Similarly, failure to develop mature biliary cells in liver organoids from an Alagille syndrome patient mirrored the biliary defects observed in patients (Huch et al., 2015).

When cerebral organoids were first established, they were also applied to the study of primary microcephaly, a genetic condition caused by a mutation in *CDK5RAP2* (Lancaster et al., 2013). The brain organoids generated from patient-derived iPSCs were overall much smaller and the individual cortical regions were especially hypoplastic. A series of observations at various time points and specific examination of mitotic spindle orientation during progenitor divisions revealed that the patient neural stem cells began dividing asymmetrically and generating neurons too early, which led to a depletion of the progenitor pool and an eventual decrease in overall neuron number. Because mice could not fully recapitulate the extent of brain-size reduction seen in humans, the organoids revealed certain morphological differences that could only be seen in this human-specific model system.

A modification of the more regionally specified telencephalic protocol summarized above (Eiraku et al., 2008) was also used to model a human neurodevelopmental condition, idiopathic autism spectrum disorder (ASD). Mariani et al., 2015 established iPSC lines from four ASD patients along with closely related unaffected controls. These were initially grown as 3D aggregates, followed by plating of rosettes as described previously (Eiraku et al., 2008; Mariani et al., 2012), with the modification that rosettes were then lifted off and grown again as 3D aggregates to yield forebrain organoids. Although generally very similar between probands and controls, the ASD organoids displayed an increased number of inhibitory interneurons as a result of upregulated FoxG1, an important factor in forebrain patterning. More recently, a genetic condition causing lissencephaly (smooth brain) was modelled using forebrain organoids, revealing defects in progenitors, including mitosis timing, spindle orientation, and defective Wnt signalling (Bershteyn et al., 2017; Iefremova et al., 2017).

Leber congenital amaurosis is a ciliopathy that affects the retina and leads to inherited blindness. To model this condition, Parfitt et al. (2016) generated retinal organoids from iPSCs with a mutation in *CEP290*, a known genetic cause of this condition. These organoids displayed normal initial development into optic cups, but the resulting tissues showed decreased ciliation and reduced cilia lengths. By restoring the expression of full-length *CEP290*, the authors were able to rescue the cilia length and protein trafficking in the cilium.

### Acquired diseases

Not only can organoids be used to model congenital conditions from stem cells of patients carrying the genetic mutation, but they can also be used to model acquired diseases such as those carried by infectious agents or acquired mutations as in the case of cancer.

#### Cancer organoids

Since the first report that HeLa cells could be grown *in vitro*, researchers have established cancer cell lines for the majority of tumour types that have facilitated seminal discoveries in cancer biology. In addition, their ease of culture have made them excellent for large-scale drug screening and development (Alley et al., 1988), and have enabled the identification of genomic markers of drug sensitivity (Garnett et al., 2012). However, they have certain drawbacks: (1) they lack the tissue architecture of the organ in question, which is often intimately related to differentiation and disease progression, (2) their establishment usually requires a strong cellular selection and (3) they present extensive heterogeneity between labs, with marked differences in gene expression and proliferation, and a considerable variability in drug responses (Ben-David et al., 2018; Hynds et al., 2018). In an attempt to obtain better models that recapitulate the architecture, genetics and drug responsiveness of patients' tumours, patient-derived xenografts (PDXs) emerged as a very suitable alternative. Unfortunately, they are not applicable to all cancer models, are expensive and are impractical for large drug screenings. [For an extended review on PDX models, see Hidalgo et al. (2014).] In that regard, the discovery that healthy tissue from colon (Jung et al., 2011; Sato et al., 2011) and stomach (Barker et al., 2010) could be expanded *in vitro* prompted many researchers to invest significant effort in using organoid technology to model cancer for personalized medicine, drug testing and drug discovery. Indeed, the use of organoids to model cancer *in vitro* has become of great interest to the cancer field, and organoids derived from tissue resections, biopsies or even circulating tumour cells have now been established (see Table 2 for a detailed list). In all cases, cancer-derived organoids more faithfully maintain the genetic and phenotypic features of the tumour of origin. In that sense, they resemble PDXs, but with the advantage of a higher establishment success rate, can be easily expanded *in vitro* and are amenable for drug screening (Weeber et al., 2017; Pauli et al., 2017).

**Table 2.**
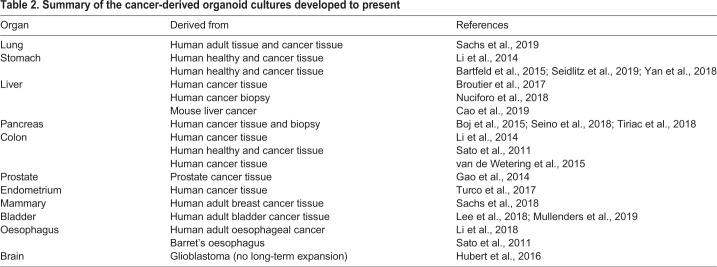
**Summary of the cancer-derived organoid cultures developed to present**

The ability to expand primary cancer tissue in a dish has opened up the possibility of living biobanks of cancer-derived organoid cultures from different tumour types, including colorectal (van de Wetering et al., 2015), gastric (Yan et al., 2018), breast (Sachs et al., 2018) and bladder (Lee et al., 2018; Mullenders et al., 2019) cancer. These provide opportunities for drug screening as well as drug development. Hence, organoids from many tissues, ranging from the ones listed above to liver (Broutier et al., 2017) and oesophageal (Li et al., 2018) cancer, have proven suitable for large drug-screening tests. While maintenance of genotypic and phenotypic features as well as suitability for drug testing does inform us about the translational potential of cancer organoids, it would be even more informative to investigate their predictive value in correlating drug-sensitivity data with clinical or genomic data. Up to today, only two studies, one in colon (Vlachogiannis et al., 2018) and the other in bladder (Lee et al., 2018) cancer organoids, have demonstrated the potential of organoids for predicting patient response. We envision that these are only the tip of the iceberg of many more studies to come to correlate the predictive value of organoids with clinical outcome.

#### Organoids as models for infectious diseases

Organoids have proven a good model to study infectious diseases and the mechanisms behind human-specific infectious agents. Hence, human small-intestinal organoids have been used to model norovirus (HuNoV) infection and propagation, and have enabled the identification of bile as a critical factor for HuNoV replication (Ettayebi et al., 2016). Similarly, *Cryptosporidium* has been shown to infect and complete its life cycle in intestinal and lung organoids (Heo et al., 2018), which has facilitated the identification of not only type II but also type I interferon signalling as a response to parasite infection. The ability to use human intestinal organoids to propagate coronaviruses *in vitro* has enabled the identification of the small intestine as an alternative infection route for Middle East respiratory syndrome coronavirus, which causes severe human respiratory infections (Zhou et al., 2017a). Similarly, gastric organoids have been used to develop models of *Helicobacter pylori* infection (Bartfeld et al., 2015; McCracken et al., 2014), while lung organoids have modelled influenza virus infection *in vitro* (Zhou et al., 2018). For extended reviews, see Bartfeld (2016), Lanik et al. (2018) and Dutta et al., 2017.

Along the same lines, cerebral organoids that model genetic microcephaly were recently adapted by a number of research groups to study the mechanisms of microcephaly caused by Zika-virus infection. This area of research has highlighted the power of brain organoid methods and the ease with which independent investigators could adopt the technology. Remarkably, initial observations of the effect of Zika on brain organoids were described as soon as 3 months after the World Health Organization (WHO) declared Zika a global health emergency in 2016. These initial reports (Garcez et al., 2016; Cugola et al., 2016; Qian et al., 2016) described overall smaller sizes of infected organoids, consistent with the microcephaly seen in patients, and a specific effect on neural progenitors, leading to cell death, reduced proliferation and premature differentiation. More recently, these Zika-infected brain organoids have been used by at least five independent groups to test treatment strategies that would prevent the effects of Zika virus infection on neural progenitors (Watanabe et al., 2017; Zhou et al., 2017b; Sacramento et al., 2017; Xu et al., 2016; Li et al., 2017). The rapidity with which a completely new disease model has been established and used for drug testing speaks to the power and future potential of these *in vitro* models for a variety of disorders.

### Gene editing in organoids to improve understanding of human diseases

The discovery of CRISPR/Cas9 gene editing as a user-friendly genetic-engineering tool compared to TALEN or zinc-finger nuclease technologies has prompted many investigators to adapt it to almost any cell type in any organism (for extended review see Adli, 2018; Sander and Joung, 2014). The Clevers lab pioneered the application of CRISPR/Cas9 technology to organoid cultures and used it to correct mutations and restore the chloride channel function of CF-patient-derived intestinal organoids (Schwank et al., 2013).

After this seminal paper, many others have highlighted the technology's applicability to organoid cultures and its relevance to further our understanding of human pathologies (see Driehuis and Clevers, 2017 for an extended review). In particular, gene editing in colon organoids has enabled the step-wise recapitulation of tumorigenesis *in vitro* (Drost et al., 2015; Matano et al., 2015), the identification of cancer signatures in microsatellite-unstable tumours (Drost et al., 2017), the discovery of new genes involved in liver cancer (Artegiani et al., 2019) or even the development of the first human brain organoid cancer model for primitive neuroectodermal tumours (Bian et al., 2018).

### Future directions and potential therapeutic applications of organoids

While organoids are powerful tools for modelling human organogenesis, homeostasis, injury repair and disease aetiology, the technology needs to overcome many hurdles to reach the next level in modelling human disease. Specifically, because of their 3D nature, the size of all organoids is limited by nutrient supply; because organoids lack vasculature, their development and growth depend upon diffusion from the surrounding media. While this is sufficient for smaller organoids or those without complex stratification, such as the branched ductal organoids and the epithelial tissues of the endoderm, the thicker tissues of the brain experience dramatic necrosis in the organoid interior. Thus, extensive effort will likely focus on improving nutrient supply and even vascularization. Some organoids have been implanted into highly angiogenic sites in rodents to enable vascularization and blood perfusion by the host (Takebe et al., 2015). This approach has even revealed that certain human-specific bi-products, for example liver metabolites, are detectable in the rodent host blood (Huch et al., 2015; Takebe et al., 2013; Hu et al., 2018). Furthermore, brain organoids transplanted into a highly angiogenic site of the rodent brain could be vascularized to improve survival and attract host-derived microglia (Mansour et al., 2018). Finally, recent work applying fluid flow to kidney organoids revealed the ability of endogenous endothelial cells to form a vascular network and improve the maturation of the kidney tissue (Homan et al., 2019). This vascularization approach has the potential to overcome tissue growth/survival limitations while maintaining *in vitro* accessibility and scalability.

Another hurdle to disease modelling and drug testing is the scalability of organoid cultures. Because of their 3D nature and complex morphologies, organoids are typically examined using laborious and time-consuming assays such as immunohistochemistry. Furthermore, their culture requires larger culture vessels and volumes of media such that it is usually quite difficult to perform drug testing in the commonly used 384-well plate format. Thus, other scaling approaches are being developed, such as mini-spinning bioreactors, or Spin-Ω, for scaling up production of brain organoids (Qian et al., 2016).

The complexity that arises from their self-organization also introduces some unpredictability to this model system, particularly in the case of human PSC-derived organoids. Thus, a desired tissue identity is not always reproducible, and even when it is present, it rarely (if ever) arises in the same configuration from organoid to organoid. This means that researchers using these methods must carefully control which organoids to use for analysis and must examine many organoids in order to separate the real phenotype from the noise of inhomogeneity. However, a number of new methods have recently been introduced to begin to address this issue, such as bioengineering methods using scaffolds or micropatterned substrates that help guide the development of stem cells to particular identities and morphologies (Warmflash et al., 2014; Lancaster et al., 2017; Knight et al., 2018).

As discussed above, organoids have proven amenable for drug testing with relatively small catalogues of drug compounds. More large-scale drug testing to identify novel compounds that may treat a range of patients will require scaling up of organoids, and the pharmaceutical industry is beginning to investigate this approach. However, in the more immediate term, organoids could be combined in the already established arsenal of 2D and bioengineered disease models. For example, cells generated from organoids could be isolated and cultured in 2D as a more accessible and scalable source. Furthermore, organ-on-a-chip approaches may be applied to organoids, or cells isolated from organoids, in order to capture the cellular diversity, but in a defined configuration. Microfluidics can also be applied to organoids to introduce fluid flow or restrict their growth in defined spatial conformations (Karzbrun et al., 2018). Finally, even with further improvements, organoids will not replace existing models in drug development, and should be thought of as complementary to other methods. In particular, organoids cannot replace animal models as the *in vivo* whole-organism context will remain a necessity when evaluating a particular drug candidate.

Not only could disease model organoids be used for drug testing, but there is an increasing use of liver organoids and liver-on-a-chip models to test drug metabolism (Kimura et al., 2018). Since the US Food and Drug Administration (FDA) now requires drug companies to demonstrate downstream metabolites before approval, human *in vitro* liver models allow for initial testing before testing in patients, in which potentially unpredicted metabolites could do harm.

Finally, while still far from being a reality, a long-term goal of organoid technologies will be to apply them to cell replacement or even whole-organ transplantation. Currently, organ donors are in short supply and many patients fail to receive a vital organ transplant in time. A source of functioning cells or tissues that could replace the faulty ones in a patient would be a major advance and potentially save thousands of lives each year. While vast improvements on the technology are still required to achieve this goal, we envision that the field might see this type of application, and many others, in the future.
